# Safety and efficacy of dihydroartemisinin-piperaquine versus artemether-lumefantrine in the treatment of uncomplicated *Plasmodium falciparum *malaria in Zambian children

**DOI:** 10.1186/1475-2875-10-50

**Published:** 2011-02-28

**Authors:** Michael Nambozi, Jean-Pierre Van Geertruyden, Sebastian Hachizovu, Mike Chaponda, Doreen Mukwamataba, Modest Mulenga, David Ubben, Umberto D'Alessandro

**Affiliations:** 1 Department of Clinical Sciences, Tropical Disease Research Center, P.O Box 71769, Ndola Zambia; 2 International Health Unit, Universiteitsplein 1,BE-2610 Antwerpen (Wilrijk), Antwerp University, Antwerp, Belgium; 3 Medicines for Malaria Venture, P.O Box 1826, 20, rte de Pré-Bois, 1215 Geneva 15, Switzerland; 4 Department Parasitology, Institute of Tropical Medicine, Antwerp, Belgium

## Abstract

**Background:**

Malaria in Zambia remains a public health and developmental challenge, affecting mostly children under five and pregnant women. In 2002, the first-line treatment for uncomplicated malaria was changed to artemether-lumefantrine (AL) that has proved to be highly efficacious against multidrug resistant *Plasmodium falciparum*.

**Objective:**

The study objective was to determine whether dihydroartemisinin-piperaquine (DHA/PQP) had similar efficacy, safety and tolerability as AL for the treatment of children with uncomplicated *P. falciparum *malaria in Ndola, Zambia.

**Methods:**

Between 2005 and 2006, 304 children (6-59 months old) with uncomplicated *P. falciparum *were enrolled, randomized to AL (101) or DHA/PQP (203) and followed up for 42 days. Outcome of treatment was defined according to the standard WHO classification, i.e. early treatment failure (ETF), late clinical failure (LCF, late parasitological failure (LPF) and adequate clinical and parasitological response (ACPR). Recurrent infections were genotyped to distinguish between recrudescence and new infection.

**Results:**

No ETF was observed. At day 28, PCR-uncorrected ACPR was 92% in the DHA/PQP and 74% in the AL arm (OR: 4.05; 95%CI: 1.89-8.74; p < 0.001). Most failure were new infections and PCR-corrected ACPR was similar in the two study arms (OR: 0.69; 95%CI: 0.22-2.26; p = 0.33). Similar results were observed for day 42, i.e. higher PCR-uncorrected ACPR for DHA/PQP, mainly due to the difference observed up to day 28, while the PCR-corrected ACPR was similar: DHA/PQP: 93% (179/192), AL: 93% (84/90), (OR: 0.92; 95%CI: 0.30-2.64; p = 0.85). Except for cough, more frequent in the DHA/PQP arm (p = 0.04), there were no differences between treatment arms in the occurrence of adverse events. Two serious adverse events were probably associated to AL treatment.

**Conclusion:**

DHA/PQP was as efficacious, safe and well tolerated in treatment of uncomplicated malaria as AL, though in the latter group more new infections during the follow up were observed. DHA/PQP seems a potential candidate to be used as an alternative first-line or rescue treatment in Zambia.

**Trial Registration:**

ISRCTN16263443, at http://www.controlled-trials.com/isrctn

## Background

In Zambia, chloroquine (CQ) has been for a long time the first-line treatment of uncomplicated malaria [1]. Reduced *in vivo *CQ sensitivity was first reported in 1978 [2] and CQ resistance in 1983 [3]. In 1996, sulphadoxine-pyrimethamine (SP) was recommended by the malaria control programme as rescue treatment if CQ failed [4]. Between 1996 and 2000, CQ and SP *in vivo *tests in children under the age of five conducted in eleven malaria sentinel sites showed that CQ resistance (based on 14 days follow up) was as high as 52% in some areas (range: 12.8-52.0%). In 1998, in the Western Province, parasitological resistance (RII-RIII) to CQ (7-day follow up) was 60% and that to SP 26% (14-day follow up), a figure higher than previously reported [5], raising concerns about the efficacy of the recently-introduced SP as it was the rescue treatment in case of CQ treatment failure. By 2000, some neighbouring countries like Kenya, Malawi, South Africa, Tanzania, Botswana and Zimbabwe had changed their first-line treatment for uncomplicated malaria from CQ to SP [6]. Considering the high resistance to CQ and SP and the WHO recommendation in 2001 to use artemisinin-based combination therapy (ACT) to combat drug resistant falciparum malaria [7], the Zambian Ministry of Health (MoH) decided in 2002 to adopt artemether-lumefantrine (AL) as first-line treatment for uncomplicated *Plasmodium falciparum *malaria [1,8]. AL was recommended despite concerns at that time over its use in pregnant women and children under 10 kg, the need of a fatty meal for the optimal absorption of lumefantrine, the short shelf life (2 years), its sustainability and cost. Indeed, it was estimated that the cost of AL needed to treat all malaria cases would exceed the total MoH allocation for basic health care [1]. Few alternatives were available [1] and since there were no data on AL use in Zambia, pharmacovigilance to monitor its tolerance was recommended. Nevertheless, AL was deployed as first line drug with remaining concerns whether sufficient health staff had been trained on its use and the risk of a vertically oriented approach as opposed to strengthening the health service provision. During this transition period, between 2003 and 2005, a clinical trial carried out in Ndola on adult patients showed that AL was significantly more efficacious than SP [9]. In 2005, a study comparing dihydroartemisinin-piperaquine (DHA/PQP) with AL for the treatment of uncomplicated *P. falciparum *malaria in children was carried out in Ndola. This was part of a phase III multicentre study done, besides Zambia, in four other African countries (Burkina Faso, Kenya, Mozambique and Uganda) [10]. More detailed results of the Zambian study are presented below.

## Methods

### Study site and population and design

The study (randomized and open-label) was conducted between September 2005 and May 2006 in Ndola, Zambia, where four peri-urban health centres (Chifubu, Chipulukusu, Lubuto and Masala) were identified for the recruitment of the patients. The study was sponsored by the Sigma-Tau, Industrie Farmaceutiche Riunite, Italy, and funded by Medicines for Malaria Venture (MMV). In Zambia, in 2006, malaria prevalence in children under five was on average 22% [11]. In Ndola, the malaria incidence (confirmed and suspected cases) estimated with the information collected by the health management information system (HMIS) was 44.9% (449/1,000) in 2005 and 28.0% (280/1,000) in 2009, showing a marked decline over a relatively short period [12]. In this peri-urban study area, the malaria endemicity is mesoendemic. It has a seasonal transmission which peaks between November and May.

### Study procedures

Children 6-59 months old attending the health facilities with suspected malaria were included if they fulfilled the following inclusion criteria: body weight >5 kg; microscopically confirmed *P. falciparum *mono-infection with asexual parasite densities between 2,000 and 200,000/μl; fever (axillary temperature ≥37.5°C) or history of fever in the preceding 24 h. Patients were not recruited if they met at least one of the following exclusion criteria: severe malaria or other danger signs; acute malnutrition (weight for height <70% of the median National Center for Health Statistics/WHO reference) [13,14] or any other concomitant illness or underlying disease; contra-indication to receive the trial drugs or history of treatment with any anti-malarial drug or drug with anti-malarial activity within the 14 days preceding enrolment. Patients satisfying the inclusion/exclusion criteria were enrolled if the parent/guardian signed a detailed written informed consent translated in local language, Bemba.

Patients were individually randomized according to a 2:1 (DHA/PQP:AL) scheme so as to have more patients in the DHA/PQP arm to provide better estimates for its cure rates and more cases for the integrated safety data base. A total of 304 study participants were enrolled. A randomization list was generated by an independent off site contract research organization (CRO), with each treatment allocation concealed in opaque sealed envelopes that were opened only after the patient's recruitment.

Both drugs were co-formulated, fixed-dose ACTs and they were administered under direct supervision during three consecutive days, according to the patient's body weight. AL (Coartem™, Novartis, Switzerland) was administered twice a day (at enrolment and at 8, 24, 36, 48 and 60 h) according to the following dosage: weight 5-14 kg: one tablet per dose; weight 15-24 kg: two tablets per dose; weight 25-34 kg: three tablets per dose. DHA/PQP (Eurartesim™, Sigma-Tau, Italy) was given once daily, at the standard dosage of 2.25 mg/kg and 18 mg/kg per dose of DHA and PQP, respectively, rounded up to the nearest half tablet. To facilitate the correct dosing of DHA/PQP, two formulations were used (DHA 20 mg + PPQ 160 mg and DHA 40 mg + PPQ 320 mg). In case of vomiting, a full dose was repeated if this occurred within the first half an hour or half a dose if it occurred between 30 minutes and 1 h. In agreement with the instruction of the manufacturer, AL was administered concomitantly with milk to facilitate the absorption of lumefantrine, while DHA/PQP was administered only with some water. For infants, drugs were crushed, mixed with water and administered as slurry. In order to minimize bias, treatment allocation was concealed until recruitment of the patient was completed. Both patient allocation to the different analysis populations and assessment of the primary end-point were made by staff blinded to the treatment assignment and before availability of the PCR results.

### Ethical considerations and patient safety

The study was approved by a local institutional ethics committee (Tropical Diseases Research Centre Ethics Committee) and the ethical and scientific committee of the Institute of Tropical Medicine, Antwerp, Belgium. The trial was conducted under the provisions of the Declaration of Helsinki (2002) and in accordance with Good Clinical Practices guidelines set up by the International Conference on Harmonization. A Study Steering Committee, a Data Monitoring Committee and a Clinical Development Committee were created prior to the beginning of the trial, and worked independently to harmonize and monitor the study. The trial was registered prior to the enrolment of the first patient in the International Standard Randomized Controlled Trials Register, number ISRCTN16263443, at http://www.controlled-trials.com/isrctn.

### Treatment follow-up, clinical and laboratory procedures

All children were kept at the health facility for the three-day dosing period. The mother/guardian was asked to return with the child for scheduled visits on days 7, 14, 21, 28, 35 and 42 post-treatment, or if any symptoms occurred. Field workers traced patients missing any visit. For each visit, a physical examination was performed by the study clinicians, vital signs were recorded and axillary temperature measured with an electronic thermometer. Adverse events and serious adverse events were recorded and monitored throughout the study.

The study was monitored by an external CRO who carried out visits on a monthly basis. Rescue treatment for recurrent parasitaemia was quinine 10 mg/kg orally three times a day for 7 days. All participants received a free insecticide-treated bed net at recruitment.

Capillary or venous blood was taken at every visit. Thick and thin blood films were prepared, dried and Giemsa-stained, and parasite density estimated by counting the number of asexual parasites in 200 white blood cells (WBC), assuming a standard WBC count of 8,000/μl. In addition, quality control was performed on 20% of all the slides at a central laboratory. Samples for haematology (full blood count) and biochemistry (liver and renal function) were taken at enrolment, at days 3, 28 and 42, and at any other visit if judged necessary by the clinician. For PCR analysis, three blood spots were collected on filter paper (Whatmann 3 MM) at enrolment and at any visit after day 7. Each filter paper was dried and individually stored in a plastic bag containing silica gel. All filter papers were subsequently transferred to the Institute of Tropical Medicine (Antwerp, Belgium) where centralized genotyping was conducted. Purification of DNA was conducted as previously described [15]. Three polymorphic genetic markers MSP1, MSP2 and GluRP were used to distinguish recrudescence from new infections (PCR-corrected). Recrudescence was defined as at least one identical allele for each of the three markers in the pre-treatment and post-treatment samples. New infections were diagnosed when all alleles for at least one of the markers differed between the two samples. All gels were re-read under blinded conditions by an independent expert (National Museum of Natural History, Paris, France). In addition, 20% of the filter papers were re-analysed and assessed by an independent laboratory (Shoklo Malaria Research Unit, Mae Sot, Thailand).

### Outcome classification

Treatment outcome was established according to standard WHO classification [16]: Early Treatment Failure (ETF) was defined as one of the following: i) danger signs or severe malaria on days 1, 2 or 3 with parasitaemia; ii) parasite density at day 2 greater than at day 0; iii) parasitaemia on day 3 with axillary temperature ≥37.5°C and iv) parasite density at day 3 equal or greater than 25% of that at day 0. Late Clinical Failure (LCF) was defined as danger signs or severe malaria or parasitaemia with axillary temperature ≥37.5°C between day 4 and day 28 (or 42), without having been previously classified as ETF. Late parasitological failure (LPF) was defined as the reappearance of parasitaemia between day 4 and day 28 (or 42) without fever and without previously meeting any of the criteria for ETF or LCF. An adequate clinical and parasitological response (ACPR) was defined as the absence of parasitaemia by day 28 (or 42) without previously meeting any of the criteria for ETF, LCF and LPF. The number of cases of total treatment failure (TTF) was computed as ETF+LCF+LPF. More detailed analysis of the outcome classification can be found in Table 1.

**Table 1:** Day 28 and Day 42 uncorrected ACPR (steps 1-11) and PCR-corrected ACPR (steps 1-16) in the different populations of analysis

Step	Event to be assessed	Per Protocol	Pure ITT
1	Withdrawal BEFORE OR AT D28: any reason except lost to follow-up	Depending on reason, a patient can be excluded or evaluated as Failure	Failure

2	Withdrawal BEFORE OR AT D28: lost to follow-up	Excluded	Failure

3 *	Withdrawal AFTER D28: any reason except lost to follow-up	Failure	Failure

4 *	Withdrawal AFTER D28: lost to follow-up	Failure	Failure

5	ETF, LCF, and LPF in accordance with the WHO criteria	Failure	Failure

6**	Presence of major protocol violations	Excluded	No effect

7**	Occurrence of adverse events highlighting recurrence of malaria	Failure	Failure

8**	Presence of missing parasitaemia at two or more consecutive scheduled visits or presence of an isolated missing parasitaemia not preceded and followed by a negative parasitaemia	Failure	Failure

9**	Administration of drugs with a known or suspected anti-malaria action as rescue treatment	Failure	Failure

10**	Administration of drugs with a known or suspected anti-malaria action as non rescue treatment	Excluded	Failure

11**	Administration of anti-malarial drugs for Plasmodium vivax, Plasmodium malariae, or Plasmodium ovale during the course of the study in patients not classified as ETF or LTF	Failure with new infection	Failure with new infection

12	PCR not done BETWEEN DAY 4 AND DAY 13	Recrudescence	Recrudescence

13	PCR: non interpretable or missing or not done BETWEEN DAY 14 AND D28	Excluded	Recrudescence

14**	PCR: non interpretable or missing or not done AFTER D28	Rule ***	Recrudescence

15	PCR = new infection or uncorrected ACPR = Failure with new infection	Success	Success

16	PCR = recrudescence	Recrudescence	Recrudescence

* For the Day 42 endpoint

** All such cases were individually revised at the Blind Data Review meeting. Protocol violations were pre-defined

*** Patients for whom the PCR is not interpretable or missing will be assigned the result "recrudescence" or "new infection" according to the ratio between these at the corresponding time point and within each treatment group, separately considered

### Statistical analysis

The primary endpoint was the PCR-corrected adequate clinical and parasitological response (ACPR) at day 28; secondary efficacy outcomes included PCR-corrected cure rates at day 42, PCR-uncorrected cure rates at days 28 and 42; parasite and fever clearance times, presence and clearance of gametocytes, and haemoglobin (Hb) recovery from baseline to day 28. All standard safety outcomes such as incidence of adverse events, changes from baseline on haematology and clinical chemistry parameters and vital sign variation during the study were also evaluated. The treatment outcome was analysed as per protocol analysis based purely on the standard definitions of early/late clinical and parasitological failure [16] (Table 1). All cases not strictly matching the WHO definitions and/or the described procedure were reviewed individually at the data review meetings.

Serious Adverse Events (SAE) were defined using the ICH GCP guidelines as any untoward medical occurrence that at any dose resulted in death, was life threatening, required hospitalization or prolongation of existing hospitalization, resulted in persistent or significant disability/incapacity.

This is a sub-analysis of a multicentre study designed as a non-inferiority trial. This study, being part of a multicenter clinical trial, was not powered to compare treatments within Zambia. All randomized patients fulfilling the protocol eligibility criteria, having taken at least 80% of the study medication, having completed the day-28 assessment and having an evaluable PCR in case of recurrent parasitaemia were included. All drop-outs and all patients with missing or non-interpretable PCR results were excluded from the PP population. Data was entered using SAS programme. Statistical analysis was done using Epi Info software version 3.4.3 and STATA statistical analysis software package (version 10; Stata Corp.). An univariate analysis was done to characterize the sample of patients in the study. To determine the risk of treatment failure, proportions were compared using the χ2 or Fisher's exact test. A two sided p-value of ≤0.05 was defined as statistical significant. In the proportional hazard regression, all patients were censored at the time of the last recorded visit and the ITT definition was used to define failure or ACPR (Table 1). The risk of new infections was compared to the other outcomes.

## Results

### Baseline characteristics and trial profile

**Figure 1:**
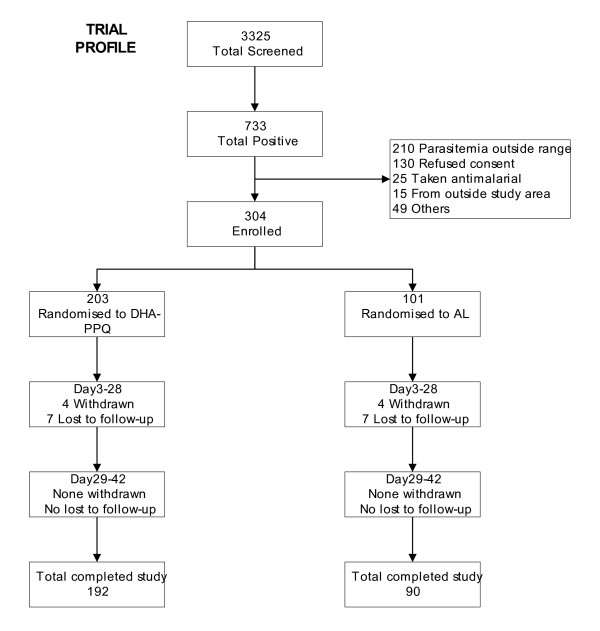
**Trial profile**.

Among 3,325 potential patients attending the 4 health facilities, 733 were within the required age, had fever (or history of fever) and peripheral parasitaemia by microscopy. Nevertheless, 429 were not included because the parasite density was outside the required range (49.0%), refused to take part in the study (30.3%), had taken an anti-malarial before screening (5.8%), were coming outside the study site (3.5%) or other (11.4%) (Figure 1). In total, 304 patients were enrolled, 203 were in the DHA/PQP arm and 101 in the AL arm. At enrolment, the two groups had similar demographic and clinical characteristics (Table 2). Out of the 304 study participants enrolled, 290 (95.4%) completed the follow-up until day 42. The percentage of patients lost to follow up was statistically similar between the two study arms respectively 3.5% (7/203) in the DHA/PQP and 7.0% (7/101) in the AL arm (p = 0.08). Parents of one patient in the DHA/PQP arm withdrew consent, 3 in the DHA/PQP arm and 4 in the AL arm were excluded from the analysis after having wrongly received an anti-malarial drug during follow up in absence of a microscopically confirmed malaria infection or after vomiting twice the study medication. In the end 282 patients, 192 in the DHA/PQP arm and 90 in the AL arm, were used for the per protocol analysis.

**Table 2:** Baseline characteristics of patients by treatment

Characteristic	DHA/PQP	AL
**Number of patients**	203	101
**Number of females (%)**	86 (42.4)	42 (41.2)
**Mean weight in kg (±SD)**	11.2 (2.5)	11.7 (2.7)
**Mean age in months (±SD)**	29.0 (14.1)	31.3 (13.8)
**Median parasite density/μL (range)**	35840 (14680-77520)	39120 (16600-86400)
**Mean haemoglobin in g/dL (±SD)**	8.9 (1.7)	9.2 (1.8)

The differences in the baseline characteristics between the treatment groups are not significant

### Clinical and parasitological outcomes

No ETF was observed. At day 14, the ACPR for both arms was 100%. At day 28, the percentage of patients with recurrent infection was significantly lower in the DHA/PQP (7.8%, 15/192) as compared to the AL group (25.6%, 23/90) (OR: 0.25; 95%CI: 0.11-0.53; p < 0.001). This difference was seen for both the LCF (3.1% *vs*. 11.1%; OR: 0.26; 95%CI: 0.08-0.80; p = 0.007) and LPF (4.7% *vs*. 14.4%; OR: 0.29; 95%CI: 0.11-0.77; p = 0.004) (Table 3). Therefore, the PCR-uncorrected ACPR was 92.2% in the DHA/PQP and 74.4% in the AL arm (OR: 4.05; 95%CI: 1.89-8.74; p < 0.001). However, according to the PCR analysis, most treatment failures were due to new infections and were significantly less frequent in the DHA/PQP group (3.1%, 6/192) as compared to the AL (18.9%, 17/90) (OR: 0.14; 95%CI: 0.05-0.39; p < 0.001). Recrudescence was low, 4.7% (9/192) *vs*. 6.7% (6/90) for DHA/PQP and AL, respectively, and not statistically different between the two study arms (OR: 0.69; 95% CI: 0.22-2.26; p = 0.33).

**Table 3:** PCR-adjusted and unadjusted treatment outcomes by days 28 and 42

	DHA/PQP (N = 192) n (%)	AL (N = 90) n (%)	Odds Ratio (95% CI)	p-value
**PCR-unadjusted**				

**Day 28**				

LCF	6 (3.1)	10 (11.1)	0.26 (0.08-0.80)	0.007
LPF	9 (4.7)	13 (14.4)	0.29 (0.11-0.77)	0.004
ACPR	177 (92.2)	67 (74.4)	4.05 (1.89-8.74)	<<0.001

**Day 42**				

LCF	11 (5.8)	13 (14.4)	0.36 (0.41-0.90)	0.014
LPF	31 (16.1)	20 (22.2)	0.67 (0.34-1.32)	0.217
ACPR	150 (78.1)	57 (63.3)	2.07 (1.15-3.71)	0.009

**PCR-adjusted**				

**Day 28**				

LCF	0 (0)	3 (3.3)		
LPF	9 (4.7)	3 (3.3)		
ACPR	183 (95.3)	84 (93.3)	1.45 (0.44-4.64)	0.33*

**Day 42**				

LCF	4(2.1)	3(3.3)		
LPF	9(4.7)	3(3.3)		
ACPR	179 (93.2)	84 (93.3)	0.98 (0.32-2.90)	0.974

*Fisher exact p values

At day 42, the percentage of patients with a recurrent infection was lower in the DHA/PQP (21.9%, 42/192) than the AL group (36.7%, 33/90) (OR: 0.48; 95%CI: 0.27-0.87; p = 0.009). Such difference was mainly due to a lower proportion of LCF in the DHA/PQP group compared to AL group (5.8% *vs*. 14.4%; OR: 0.36; 95%CI: 0.14-0.90; p = 0.014), while the proportion of LPF did not differ between the two study arms (16.1% *vs*. 22.2%; OR: 0.67; 95%CI: 0.34-1.32; p = 0.22). As for the estimation at day 28, most recurrent infections were new infections, significantly less in the DHA/PQP (15.1%, 29/192) than in the AL (30.0%, 27/90)group (OR: 0.42; 95%CI: 0.22-0.79; p = 0.003) (Table 3). Such difference occurred mainly within the first 28 days as afterwards the occurrence of new infections was similar between the two study groups (12.0% *vs*. 11.1%). Only four recrudescences were observed after day 28, all of them in the DHA/PQP group. The PCR-corrected ACPR was similar between the two study groups: DHA/PQP: 93.2% (179/192), AL: 93.3% (84/90), (OR: 0.92; 95%CI: 0.30-2.64; p = 0.85).

Parasite clearance was rapid in both treatment groups (Kaplan-Meier estimate of median time was 2 days in each group, in both populations). About 60% of patients had fever at baseline while at day 2 more than 97% of patients were afebrile in both treatment groups. Gametocyte prevalence at recruitment was similar in both study arms (ITT: DHA-PQP 11.75%; AL 12.94%, p = 0.501; PP: DHA-PQP 11.55%; AL 13.36%, p = 0.326). However, gametocyte carriage measured as rate of person-gametocyte-weeks was significantly higher in the DHA-PQP group than in the AL group, both for the ITT (DHA-PQP: 43.97/1,000; AL: 21.43/1,000; p = 0.005) and the PP (DHA-PQP: 42.65/1,000; AL: 21.23/1,000; p = 0.006) populations.

In patients with no recurrent parasitemia, hemoglobin was increased at day 28 in both DHA-PPQ arm (+1.39 g/dL; 95%CI: 1.13-1.66; p < 0.0001) and AL (+0.89 g/dL; 95%CI: 0.48-1.31; p < 0.0001) and was different between treatment groups (p = 0.047). In patients with no recurrent parasitemia, hemoglobin was increased at day 42 in both DHA-PPQ arm (+1.91 g/dL; 95%CI: 1.23-1.86; p < 0.0001) and AL (+1.45 g/dL; 95%CI: 0.95-1.71; p < 0.0001) and was not different between treatment groups (p = 0.45).

**Figure 2:**
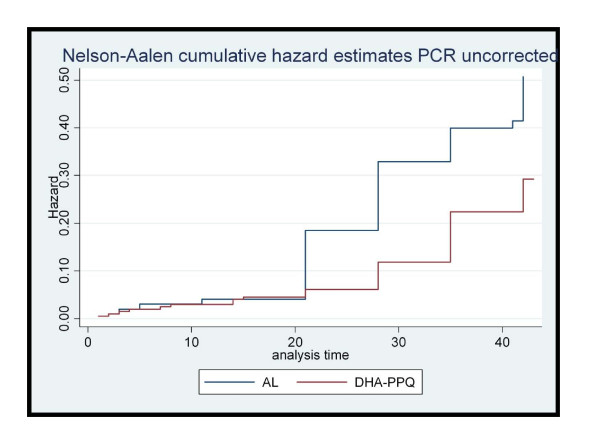
**Cumulative Hazard estimates for PCR-unadjusted treatment failure on day 42 by treatment group, Zambia, 2009**.

**Figure 3:**
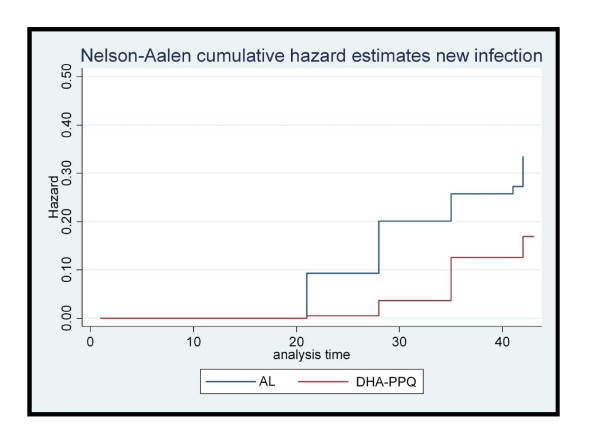
**Cumulative Hazard estimates of re-infection by day 42 by treatment group, Zambia, 2009**.

**Figure 4:**
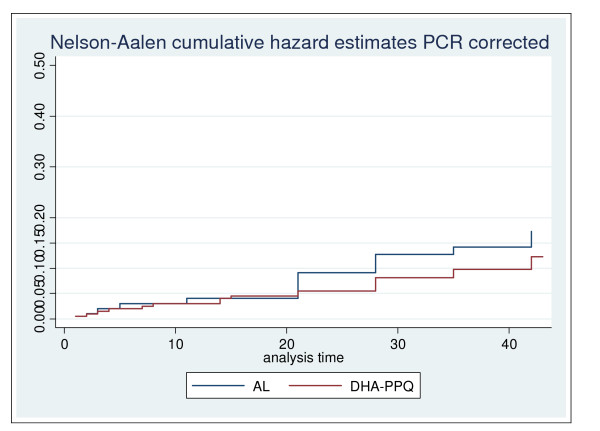
**Cumulative Hazard estimates of recrudescence by day 42 by treatment group Zambia, 2009**.

At day 28 the survival analysis, using the ITT definition, showed a hazard Ratio (HR) (DHA/PQP/AL) of 0.35 for PCR-uncorrected treatment failure (95%CI: 0.21-0.62; p < 0.0001) (Figure 2), 0.17 for new infections (95%CI:0.07-0.43; p < 0.0001) (Figure 3) and 0.60 that for true failures (recrudescence) (95%CI: 0.34-1.40; p = 0.31) (Figure 4). The survival analysis till day 42 showed a HR of 0.55 (DHA/PQP/AL) for PCR-uncorrected treatment failure (95%CI: 0.37-0.82; p = 0.004) (Figure 2), 0.47 for new infections (95%CI: 0.28-0.79; p = 0.005) (Figure 3) and 0.70 for true failures (recrudescence) (95%CI: 0.36-1.34; p = 0.29) (Figure 4).

### Adverse events

Seven serious adverse events (SAE) were observed: three of them for patients (two in DHA/PQP and one in AL) having prolonged hospitalization due to late fever clearance; in addition, in the DHA/PQP group, one patient developed severe malaria and another had persistent fever, weakness and anorexia; both were treated with quinine. In the AL group, one patient developed severe anaemia on day 3, possibly associated with study drug, and was treated with quinine and blood transfusion; the other patients had jaundice on day 3, possibly associated to study drug. All patients recovered completely. No deaths were observed during the study.

Both treatments were generally well tolerated and most adverse events were associated with the disease during the initial clinical episode or to the recurrent parasitemia during the follow-up. Cough, attributed to respiratory tract infections, was significantly more frequent in the DHA/PQP arm than the AL arm while diarrhoea was of borderline significance (Table 4).

**Table 4:** Proportions of adverse events by treatment group during follow-up

	DHA/PQP (N = 199)	AL (N = 100)	p- Value
Anorexia	6.8%	8.3%	0.24
Cough	21.2%	15.4%	0.04
Diarrhoea	7.1%	3.8%	0.051
Fever	12.0%	14.4%	0.32
Respiratory tract infections	11.1%	8.7%	0.20
Vomiting	2.6%	3.8%	0.29

## Discussion

Both DHA/PQP and AL were highly efficacious in treating uncomplicated malaria in Zambian children, even if statistically significant differences in favour of DHA/PQP were observed for the uncorrected ACPR (for LPF the difference was statistically significant only at D28). In addition, the rate of recurrent infections, particularly within the first 28 days of follow up, was significantly higher in children treated with AL. One of the strengths of this study is that it was done in an area of meso-endemic malaria, in children under five, a high risk group, and had a prolonged follow-up period of 42 days not to miss late recrudescences and to reflect the terminal half-lives of the drugs [16]. One of the limitations was it was not blinded so that an influence on the evaluation of the tolerability cannot be excluded. In terms of robustness of the trial, this weakness was mitigated with a blinded randomization procedure.

In this study, patients treated with DHA/PQP had fewer re-infections, an added advantage as it allows patient haematological recovery before a new infection sets in, especially in high transmission areas. The withdrawal and lost to follow-up rate (7%) were within the WHO recommended limits [16]. There are few studies that have compared these two forms of fixed-dose ACT. One study in Tanzania reported *P. falciparum *multi-drug resistant genes, which were weakly and indirectly associated with a decreased *in vitro *susceptibility to lumefantrine [17], indicating that resistance may emerge for the long acting partner drugs of the artemisinin derivative. In one Rwandan study, DHA/PQP was well-tolerated and highly efficacious against multidrug resistant *P. falciparum *[18]. Another study in Uganda reported similar findings, with both drugs (DHA/PQP and AL) having good efficacy and tolerability [19] Similarly, in a study carried out in Papua Indonesia, half of the patients presented with recurrent parasitaemia by day 42, mostly due to re-infections [20]. The difference in re-infections in this study and most studies could be explained by the pharmacokinetic properties of piperaquine which has a longer half-life (2-3 weeks) than lumefantrine (4-10 days) [21,22]. Indeed, long acting partner drugs provide protection after initial treatment and can reduce the risk of re-infection in high transmission areas, which, per se, can be seen as a substantial help for the national health care system. Nevertheless, the persistence of the drug at sub-optimal therapeutic doses may lead to selection of drug resistant parasites [19]. Even in Ndola, where malaria is mesoendemic, the risk of new infections was high, with the potential of selecting resistant parasites [23]. Such theoretical risk, common for all forms of ACT in which one of the components is a long-lasting anti-malarial drug, may be reduced by the use of several ACTs as first-line treatment. This could be feasible as the number of ACTs available today would allow such approach. Finally, a malaria episode occurring a few weeks after treatment, be it a recrudescence or re-infection, is perceived by both the health care providers and the users (patients and families) as another attack needing another course of anti-malarials. From this point of view, employing a treatment with a long post-treatment prophylactic effect could be an advantage.

Both drugs are co-formulated but AL should be administered twice a day for three days whilst DHA/PQP is given once a day for three days. Furthermore, AL should be administered with a fatty meal for an effective absorption of lumefantrine. In Zambia, where 80% of the population is under the poverty level, the staple diet is based on maize [24], it is difficult to provide a fatty meal for patients before the intake of the drug. This could result in a lower adherence or/and sub-optimal therapeutic doses for AL compared to DHA/PQP. The unsupervised treatment of DHA/PQP and AL has already been shown to be as effective as the supervised treatment [25,26]. As this study assessed the efficacy, it would be interesting to know the effectiveness of these two drugs.

In summary, DHA/PQP may be a good alternative to AL [19], but there are still some questions in the context of public health distribution of millions of treatments, about safety (Phase IV), effectiveness, sustainability and cost of newly available forms of ACT, such as DHA/PQP [27]. Furthermore, as the post-treatment prophylaxis is most important in areas of high malaria transmission with a high re-infection rate, the deployment of these new fixed-dose combinations should not necessarily be generalized, rather done according to the local characteristics [18,19].

## Conclusion

Considering the above challenges, the direct and indirect costs, and the lessons learnt from the current treatment policy change, DHA/PQP could be employed as rescue and/or alternative treatment to AL or as second-line treatment for patients re-attending the health facility with malaria a few weeks after receiving the first line treatment. This would be a major improvement as compared to the current policy of administering quinine to any suspected case of failure, a policy against the WHO recommendations to use an alternative ACT for the second-line treatment [28]. It would also reserve quinine for the treatment of severe malaria cases. This strategy would limit the impact on the current treatment policy as DHA/PQP would be introduced smoothly into the health system without changing the first line treatment. If effectiveness and cost-effectiveness studies turn out in favour of the DHA/PQP combination, a switch between AL and DHA/PQP as first-line therapy can still be debated and implemented depending on the malaria endemicity. Whatever the case, DHA/PQP is a credible option to be included in the anti-malarial treatment policy in Zambia.

## Competing interests

The authors declare that they have no competing interests.

## Authors' contributions

All authors contributed to the design of the study and assisted with data interpretation. MN, UDA and MM coordinated the study and supervised the enrolment and follow-up of patients. JPVG participated in data entry, collection and analysis of data. All authors participated in the preparation of the manuscript and approved the final version.
